# Analysis of nondegradable cyclins reveals distinct roles of the mitotic cyclins in *Drosophila* meiosis

**DOI:** 10.1093/g3journal/jkae066

**Published:** 2024-03-29

**Authors:** Mohammed Bourouh, Rajdeep Dhaliwal, Rajni Rai, Hafsah Qureshi, Andrew Swan

**Affiliations:** Department of Biomedical Sciences, University of Windsor, Windsor, Ontario N9B 3P4, Canada; Department of Biomedical Sciences, University of Windsor, Windsor, Ontario N9B 3P4, Canada; Department of Biomedical Sciences, University of Windsor, Windsor, Ontario N9B 3P4, Canada; Department of Biomedical Sciences, University of Windsor, Windsor, Ontario N9B 3P4, Canada; Department of Biomedical Sciences, University of Windsor, Windsor, Ontario N9B 3P4, Canada

**Keywords:** *Drosophila*, meiosis, APC/C, cyclin

## Abstract

Meiosis is a complex variant of the mitotic cell cycle, and as such relies on many of the same proteins involved in mitosis, but utilizes these in novel ways. As in mitosis, Cdk1 and its cyclin partners, Cyclin A, B, and B3 are required at multiple steps in meiosis. Here, we study the effect of stabilized forms of the three mitotic cyclins to study the consequences of failure to degrade the cyclins in meiosis. We find that stabilized Cyclin B3 promotes ectopic microtubule polymerization throughout the egg, dependent on APC/C activity and apparently due to the consequent destruction of Cyclin A and Cyclin B. We present data that suggests CycB, and possibly CycA, can also promote APC/C activity at specific stages of meiosis. We also present evidence that in meiosis APC/C^Cort^ and APC/C^Fzy^ are able to target Cyclin B via a novel degron. Overall, our findings highlight the distinct functions of the three mitotic Cdk–cyclin complexes in meiosis.

## Introduction

Meiosis is a highly specialized variation on the typical mitotic cell cycle, in which two mitotic-like divisions without an intervening S-phase result in the production of four haploid gametes. The mitotic cyclins and their kinase partner, Cdk1 are responsible for many of the important steps in meiosis. In *Drosophila*, all three mitotic cyclins play a role in driving the first major transition in meiosis, nuclear envelope breakdown (Bourouh *et al*. 2016). Cyclin A is required for bi-orientation of homologues in metaphase I, and it functions redundantly with Cyclin B3 to prevent DNA replication during the meiotic divisions (Bourouh *et al.* 2016). In *Drosophila*, mature eggs remain stably arrested in metaphase I until ovulation triggers the resumption of meiosis. Cyclin B is necessary for maintaining the metaphase I arrest, possibly by inhibiting the activity of the Anaphase Promoting Complex or Cyclosome (APC/C) (Bourouh *et al.* 2016).

The APC/C ubiquitin ligase is necessary for resumption of meiosis at ovulation. In *Drosophila*, two APC/C activators, the mitotic activator Fzy (Cdc20), and the meiosis-specific, Cort, play the key role of substrate recognition. Upon full activation at ovulation, the APC/C drives completion of meiosis by targeting Securin and the mitotic cyclins for destruction (Yamano 2019). Securin destruction frees up the protease, Separase. How these APC/Cs are activated in meiosis is not yet clear, but CycB3 appears to play a key role. From *C. elegans* to mouse, CycB3 plays a key role in meiosis. In *Drosophila*, it functions redundantly with the other cyclins in mitotic cells, but it is essential for female meiosis (Jacobs *et al.* 1998). *CycB3* mutants arrest in meiosis with elevated levels of the other two mitotic Cyclins, CycA and CycB, while the expression of a nondegradable, D-box mutated form of CycB3 (CycB3^D^), leads to a dramatic loss of these cyclins as well as endogenous CycB3 (Garrido *et al.* 2020). Genetic reduction of *CycB3* leads to a proportionate decrease in the phosphorylation of APC/C subunit, APC3, and importantly, to a decrease in Cort and Fzy association with APC/C (Garrido *et al.* 2020). These results support a model for APC/C activation via a phospho-relay mechanism in which APC3 phosphorylation leads to phosphorylation of other APC/C subunits, leading to activator binding (Fujimitsu *et al.* 2016). Furthermore, these results suggest that CycB3–Cdk1 may be the kinase for APC3 in female meiosis (Garrido *et al.* 2020).

The importance of cyclin destruction in mitotic cells has been well studied. In vertebrate systems, the failure to degrade CycA leads to an anaphase arrest (Geley *et al.* 2001). Failure to degrade CycB also leads to an anaphase arrest (Holloway *et al.* 1993), or at higher levels, a metaphase arrest (that reflects Cdk1–CycB role in inhibiting Separase) (Stemmann *et al.* 2001). In *Drosophila*, the three mitotic cyclins are degraded sequentially in mitotic cells, starting with Cyclin A in prometaphase, Cyclin B at anaphase onset, and Cyclin B3 later in anaphase (Sigrist *et al.* 1995). The expression of a stabilized form of CycA leads to a delay in anaphase, while stabilized CycB and B3 result in early and later anaphase arrests, respectively (Sigrist *et al.* 1995). While cyclin destruction via APC/C is necessary for completion of anaphase in mitotic cells in several organisms, its significance in meiosis is not as well established. Injection of stabilized forms of CycB lead to a metaphase I arrest in mouse oocytes, indicating that CycB plays an essential role in homologue separation in anaphase I (Herbert *et al.* 2003). Similar experiments with stabilized CycA reveal an opposite effect—precocious sister chromatid separation in meiosis I, suggesting a role for CycA in the removal of centromeric cohesion (Touati *et al.* 2012). The effect of stabilized CycB3 in meiosis has not been determined. In *Drosophila*, expression of a stabilized form of CycB results in a variable meiotic arrest (Swan and Schupbach 2007), indicating that its destruction is necessary for proper completion of meiosis. The significance of CycA and CycB3 destruction at the completion of meiosis has not been determined in *Drosophila*. Here, we examine the effects of expression of stabilized forms of the mitotic cyclins in meiosis. We find that degradation of all three mitotic cyclins is necessary for the completion of meiosis, though the effect of each is distinct. Our findings further our understanding of how APC/C is activated in meiosis and how its targeting of the mitotic cyclins is necessary for the completion of meiosis and preparation of the egg for embryogenesis.

## Materials and methods

### Fly stocks and crosses

The following fly stocks were generated for this study, using Gateway cloning vectors that have *pUASp* and an N-terminal tag: *Venus-(Ven)-CycA*^*wt*^, *Ven-CycA*^*Δ1–53*^, *Ven-CycA*^*Δ1–170*^, *Ven-CycB*^*Δ1–53*^, *Ven-CycB^Δ1–170^*, *Ven-CycB*^*KEN*^ (KEN box changed to AAA), *Ven-CycB*^*Δ1–53,KEN*^, *Ven-CycB*^*D,K*^ (contains the KEN to AAA change and RxxLxxxxN to GxxAxxxxA as in Raff *et al.* (2002)). *GFP-CycB*^*D*^  *(GFP-CycB^TPM^)* was obtained from Jordan Raff (Raff *et al.* 2002). *GFP-CycB3*^*D*^ was described in Garrido *et al.* (2020) and *Flag-CycB3*^*D*^ is identical but with the Flag-tag. *White^0994^*, *fzy*^*0442*^, and *cort*^*0326*^ are *UASp-RNAi* lines from the TRiP collection, and were obtained from Bloomington Drosophila Stock Center (BDSC). The w*^0994^* line was used to equalize the number of *UAS* transgenes in experiments where flies expressing a single *UAS* transgene are compared to flies expressing two *UAS* transgenes. This eliminates differences in transgene expression that can result from the dilution of limiting Gal4 by two *UAS* lines. *Mat-α4-Tubulin-Gal4* (BDSC) was used to drive transgene expression for all experiments. *Cort^RH^* and *cort*^*QW*^ were obtained from BDSC. *Yw* was used as a wild-type control for Westerns and IF. For Western blotting experiments and for some phenotype analysis, we crossed females to sterile males. This ensured that, regardless of maternal genotype, embryos did not develop, thus eliminating variability that arises from comparing developing embryos to meiosis arrested eggs. Sterile males were obtained by crossing *C(1:Y)1* (BDSC) males to *yw* females. All crosses were performed at 22 °C.

### Immunofluorescence and FISH

Aged eggs as indicated were dechorionated in undiluted bleach and then washed in embryo wash (0.7% NaCl, 0.05% Triton-X). Eggs were fixed and devitellinzed in 1:1 ratio of methanol:heptane while shaking vigorously. Eggs were then stored in −20 °C, or were rehydrated and processed. Ovaries were collected by dissecting females in isolation buffer as in Guo *et al.* (2016). Ovaries were then fixed in 4% formaldehyde in PBS, 0.2% Tween-20 (PBST), and heptane (1:1) with EGTA to stabilize microtubules. Ovaries were then extracted using PBST with 0.05% Triton-X for 30 min. For late-stage oocytes, ovaries were dissected in isolation buffer and fixed in 4% formaldehyde as above. Oocytes were then transferred to 100% methanol for sonication to remove the chorion and vitelline membrane as in Guo *et al.* (2016). Following rehydration, oocytes underwent an extraction step as above. Immunostaining followed standard methods. Rat anti-Tubulin YL1/2 (Sigma) was used ½,000. DNA was stained with either Oligreen (Invitrogen) 1/10,000, or mouse anti-Histone H3 (Chemicon) used at 1/2,000. Secondary Alexa conjugated antibodies (Invitrogen) were used at 1/1,000. FISH to identify the X-chromosome was performed using a probe that recognizes a 359 bp pericentric repeat as described in Guo *et al.* (2016). Images were taken on an Olympus FV-1000 scanning confocal microscope. Images were adjusted for contrast and brightness only, using Photoshop.

### Western blotting

Late-stage oocytes were collected from ovaries dissected in isolation buffer as above supplemented with collagenase. Stage 14 oocytes were enriched by repeated rounds of rinsing with isolation buffer, and removing the slower settling smaller egg chambers. Laid eggs were collected from females in egg collection cups on apple juice agar supplemented with liquid yeast. Wild type unfertilized eggs were collected from females crossed to XO males. Western blotting followed standard procedures. Antibodies used were anti-CycA A12 at 1/5 and mouse anti-CycB F2F4 1/20 (Developmental Studies Hybridoma Bank). Mouse anti-Actin (Millipore) was used 1/5,000. Rabbit anti-PSTAIR (Santa Cruz) was used 1/1,000. Rabbit anti-GFP (Tory Pines) was used 1/1,000. Rabbit ani-CycB3 was a gift from Christian Lehner, and was used at 1/5,000. HRP-conjugated secondary antibodies (Roche) were used at 1/7,000 and detected by ECL reagents (Pierce). Alpha Innotech FluorChem HD2 imager or Protein simple FluoroChem E imager. Densitometry was performed using ImageJ. Quantification of transgenic CycB levels relative to endogenous CycB was determined from the western blots that were used for Figs. 5 and 7. Ven-CycB^wt^ (1.8× endogenous), GFP-CycB^D^ (0.9× endogenous), and Ven-CycB^KEN^ (0.9 × endogenous) were determined from the blots in Fig. 5. Levels of Ven-CycB^Δ170^ (2.6× endogenous) were determined by comparing transgene levels to that of CycB from the wild type (*yw*) lane in the same blots (Fig. 7).

**Fig. 1.**
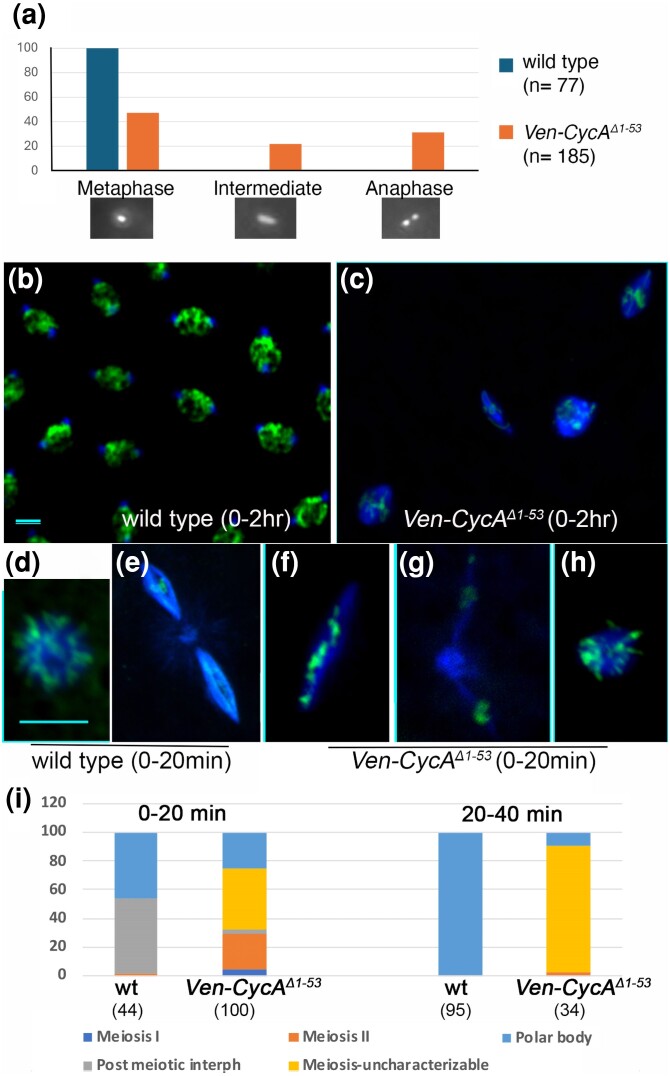
Failure to degrade Cyclin A leads to aberrant meiosis. a) Quantification of stage 14 meiotic phenotype in wild type and *Ven-CycA*^*Δ1–53*^ females (driven by *matα4-Tubulin-Gal4*). Chromatin was detected with Oligreen, and oocytes were categorized as being in metaphase I, anaphase or an intermediate state in which a single stretched chromatin mass is present. Representative images are shown below each category on the graph. b, c) Embryos from 0 to 2 h egg collections from wild type (yw) (b) and *Ven-CycA*^*Δ1–53*^ females (c) labeled for microtubules (blue) and chromatin (green). The wild-type embryo contains multiple synchronized nuclei (in this example, in prophase), while the *Ven-CycA*^*Δ1–53*^ embryo contains a small number of abnormal spindles and microtubule/chromatin arrays. d–h) Eggs from unfertilized *yw* (d, e) and *Ven-CycA*^*Δ1–53*^ females (f–h) labeled as above. d) A wild-type polar body. e) Metaphase II in wild type. f) Abnormal meiosis I in *Ven-CycA*^*Δ1–53*^. g) Abnormal meiosis II in *Ven-CycA*^*Δ1–53*^. h) Uncharacterizable microtubule/chromatin array in *Ven-CycA*^*Δ1–53*^. i) Quantification of meiotic stages in 0–20 and 20–40 min egg collections. Numbers in brackets indicate number of eggs counted.

**Fig. 2.**
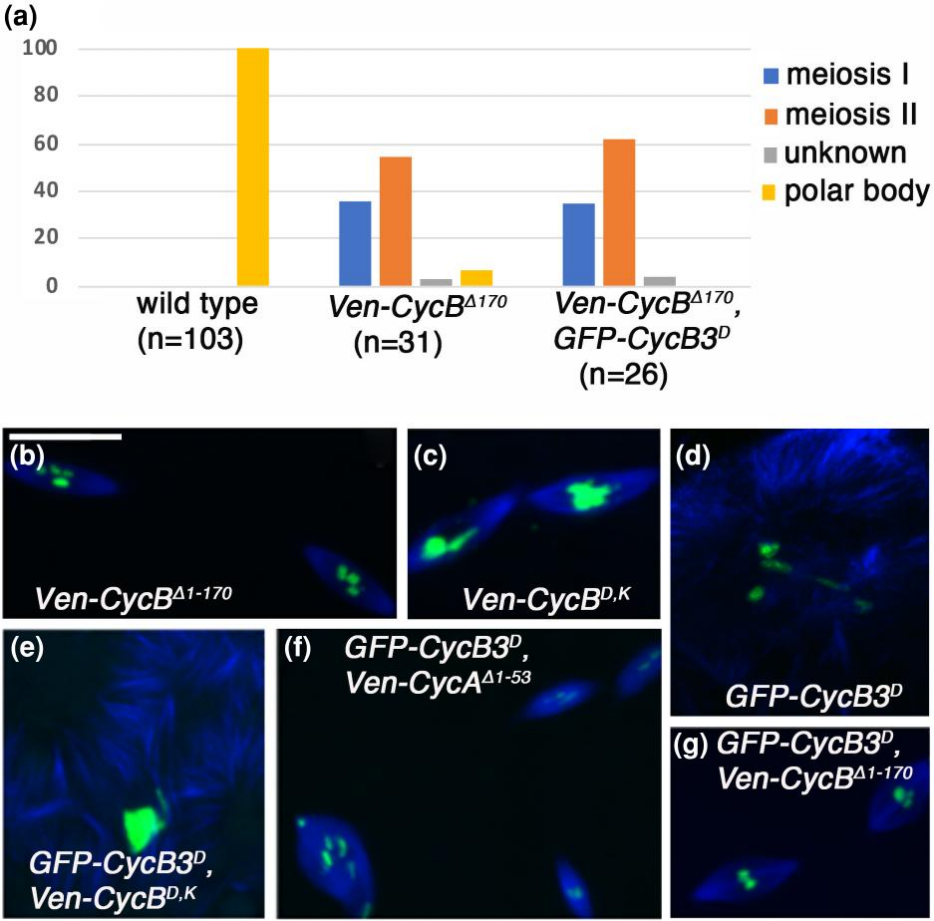
Cyclin B degradation is necessary for the completion of meiosis and for ectopic microtubule polymerization by nondegradable CycB3. a) Eggs aged 0–3 h from unfertilized wild-type females, fertilized *Venus-CycB*^*Δ1–170*^ and fertilized *Venus-CycB*^*Δ1–170*^, *GFP-CycB3*^*D*^ females classified according to the stage of meiosis observed. Meiosis I (egg contains a single spindle), meiosis II (2 spindles), polar body (1 or 2) indicates completion of meiosis. Unknown represents eggs with more than two spindles or other microtubule/chromatin masses. Number of eggs counted is indicated in brackets. b, c) *Venus-CycB*^*Δ1–170*^ and *Venus-CycB*^*D,K*^ cause a meiosis arrest (meiosis II in these examples) but no microtubule polymerization. d) Microtubule polymerization in *GFP-CycB3*^*D*^. e) *Ven-CycB*^*D,K*^ does not suppress the ectopic microtubule polymerization by *GFP-CycB3*^*D*^. *Ven-CycA*^*Δ1–53*^ (f) *and Ven-CycB*^*Δ1–170*^ (g) do suppress the *GFP-CycB3*^*D*^ ectopic microtubule polymerization phenotype.

**Fig. 3.**
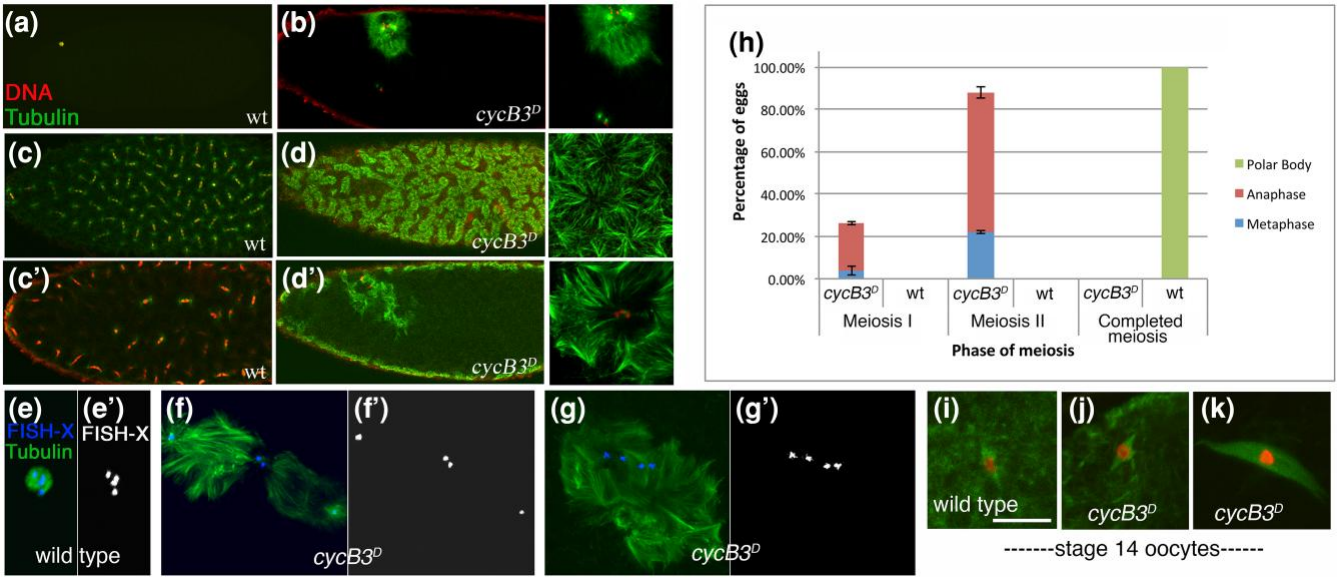
Ectopic microtubule polymerization in eggs expressing nondegradable CycB3. a–d) Fertilized eggs (0–2 h) from wild-type (wt) and *GFP-CycB3*^*D*^ females labeled for microtubules (green) and chromatin (red). a) A wild-type embryo with polar body (the zygotic nuclei are not visible in this view). b) *GFP-CycB3*^*D*^ embryo with ectopic microtubules around the female meiotic products (zoomed in view is shown on the right). c) surface and c′) transverse view of a syncytial blastoderm embryo. d) Surface and d′) transverse view of a *GFP-CycB3*^*D*^ embryo (zoomed in views shown to the right of each). The internal microtubules seen in (d′) are associated with the meiotic products, while the cortex contains further microtubule arrays. e–g) Zero to 40 min unfertilized eggs from wild-type (yw) and *GFP-CycB3*^*D*^ females probed with a X-chromosome centromeric FISH probe (blue) and for microtubules (green) FISH signal is also shown separately in grayscale (e′–g′). e) Wild type polar body with four X-chromosomes. f) *GFP-CycB3*^*D*^ egg in late anaphase with excess microtubules on the meiotic spindles. g) *GFP-CycB3*^*D*^ egg in early anaphase (as indicated by the spacing of the X-chromosomes). The meiotic spindle is not recognizable amongst all of the ectopic microtubules. h) Relative frequencies of different meiotic stages in 0–40 min wild-type and *GFP-CycB3*^*D*^ eggs. All wild-type eggs in this experiment had completed meiosis and had one or more polar bodies, while all *GFP-CycB3*^*D*^ eggs were still in meiosis. i–k) Stage 14 oocytes from wild-type and *GFP-CycB3*^*D*^ females, labeled for microtubules and chromatin. i, j) Early metaphase I spindles from wild type and *GFP-CycB3*^*D*^ appear similar, and with no evidence of ectopic microtubules. k) Mature metaphase I spindle in *GFP-CycB3*^*D*^ female, with no evidence of ectopic microtubules.

**Fig. 4.**
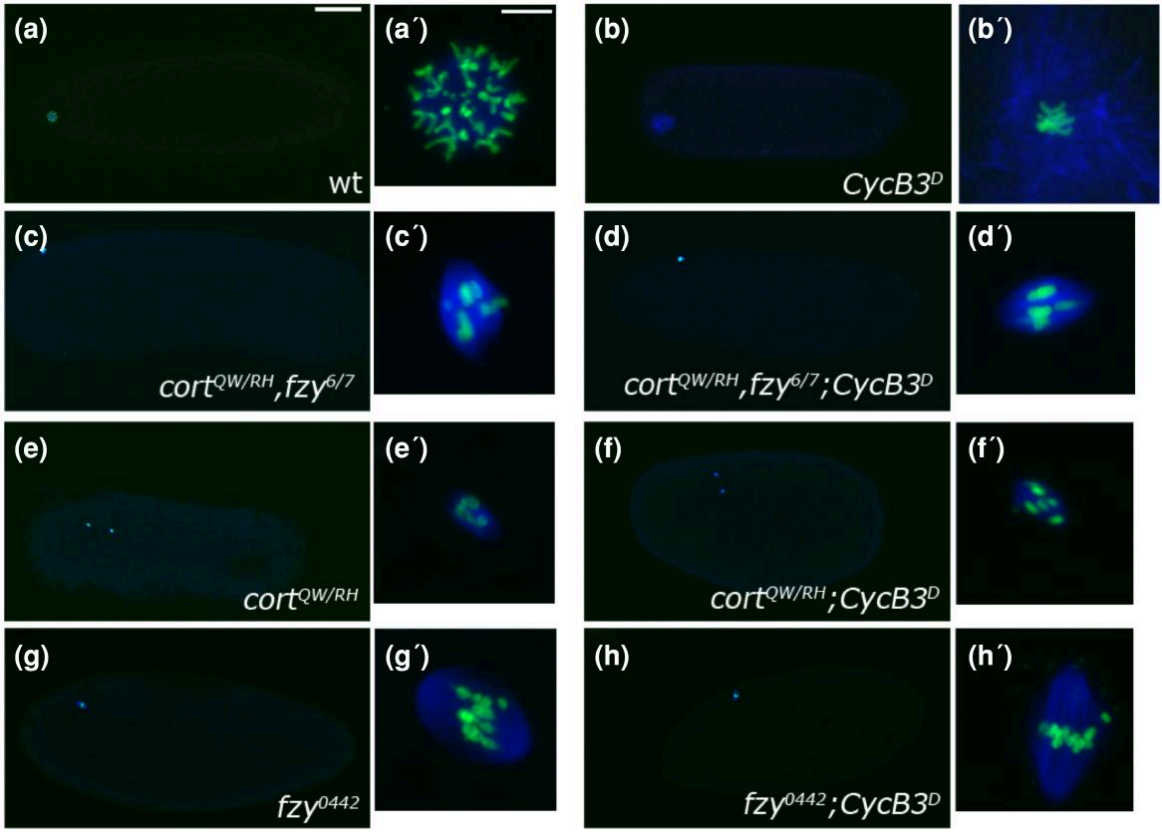
Cort and Fzy are required for ectopic microtubule polymerization in *GFP-CycB3*^*D*^ eggs. Unfertilized eggs were stained for microtubules (blue) and chromatin (green). (a)–(h) are whole eggs, (a′)–(h′) are closeups of the meiotic products. a–a′) wild-type egg with a single polar body. b–b′) *GFP-CycB3*^*D*^ egg in meiosis with ectopic microtubules. c–d′) *cort*,*fzy* double mutants and *cort*, *fzy*, *GFP-CycB3*^*D*^ show a similar meiosis arrest and absence of ectopic microtubules. e–f′) *Cort* mutant and *cort, GFP-CycB3*^*D*^ eggs both arrested in meiosis II, with two meiotic spindles, and no ectopic microtubules. g–h′) *fzy*^*0442*^ RNAi and *fzy*^*0442*^ RNAi with *GFP-CycB3*^*D*^ both arrested in meiosis I with a single meiotic spindle and no ectopic microtubules. Scale bar in a = 50 µm and applies to a–h. Scale bar in a′=5 µm and applies to a′–h″.

**Fig. 5.**
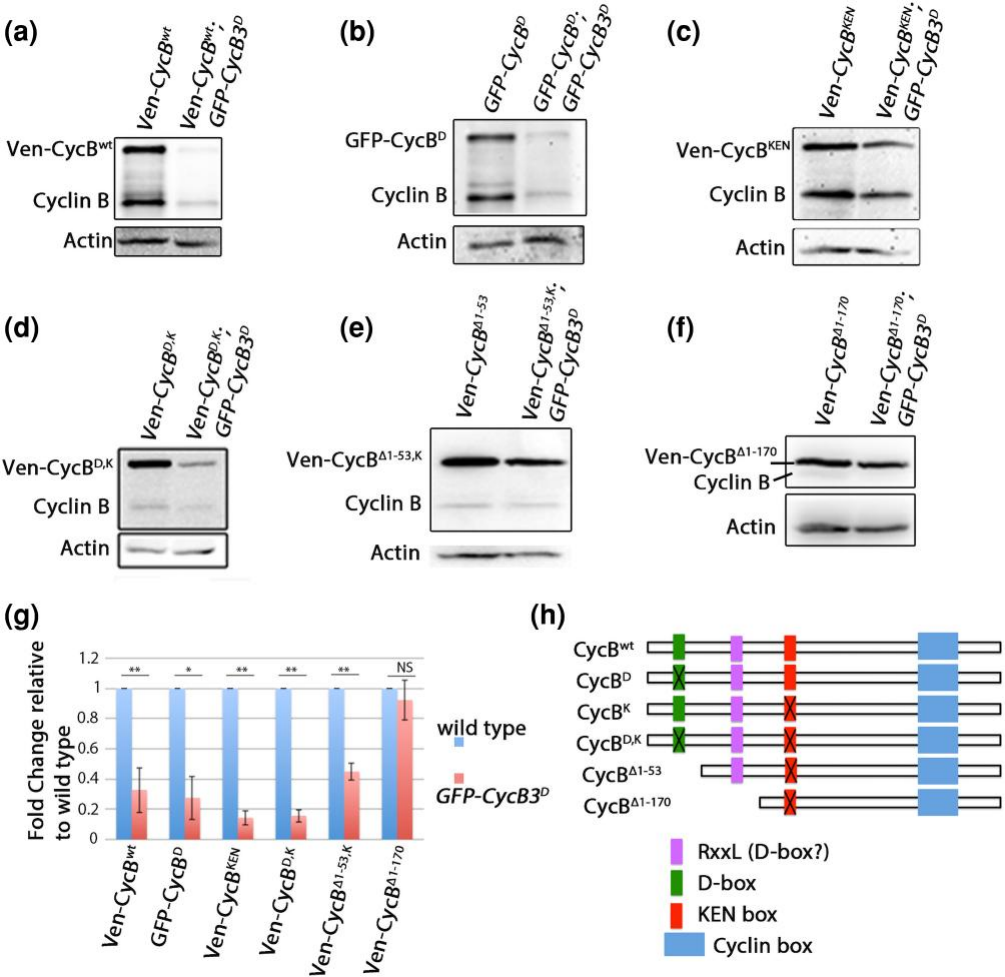
CycB3^D^ promotes degradation of full length as well as D-box and KEN-box deleted CycB transgenes. a–f) *UAS-Venus-CycB* transgenes are co-expressed with either *UAS-w^0094^* (control) or *UAS-GFP-CycB3*^*D*^ in 0–2 h unfertilized eggs, followed by Western blotting with anti-Cyclin B antibodies to detect transgenic and endogenous Cyclin B. a) Venus-CycB^wt^ levels are reduced in the presence of Flag-CycB3^D^. Endogenous CycB is similarly reduced. b, c) Presence of *GFP-CycB3*^*D*^ results in reduced levels of transgenic Venus-CycB^D^ (b) and transgenic Venus-CycB^K^ (c). Endogenous CycB is similarly reduced in both. d, e) Presence of GFP-CycB3^D^ results in reduced levels of Venus-CycB^D,K^ (d) and Venus-CycB^Δ1–53,K^ (e). In both cases, endogenous Cyclin B appears at low levels even in the absence of GFP-CycB3^D^. f) GFP-CycB3^D^ does not lead to a significant reduction of Ven-CycB ^Δ1–170^ when these are co-expressed. Endogenous Cyclin B levels appear low even in the absence of GFP-CycB3^D^. g) Quantification of Venus-CycB levels in the presence of Flag-CycB3^D^. Data was taken from at least two different experiments. Error bars represent s.e.m. Statistical significance was calculated by one tailed *T*-test, where *P < 0.05, NS, not significant. Levels of transgenic CycB relative to endogenous CycB from these blots indicate that Ven-CycB^wt^ is expressed at 1.7× endogenous Cyclin B levels. Ven-CycB^D^, GFP-CycB^D^, and Ven-CycB^K^ are all expressed at similar levels to that of endogenous CycB. Ven-CycB^D,K^, Ven-CycB^Δ1–53,K^, and Ven-CycB ^Δ1–170^ could not be quantitated relative to endogenous CycB due to their apparent effect on endogenous CycB levels (see Fig. 7). However, data from Fig. 7 allows us to determine that Ven-CycB^Δ1–170^ is expressed at approximately 2.6× normal levels of endogenous CycB. h) Schematic showing destruction motifs on CycB.

**Fig. 6.**
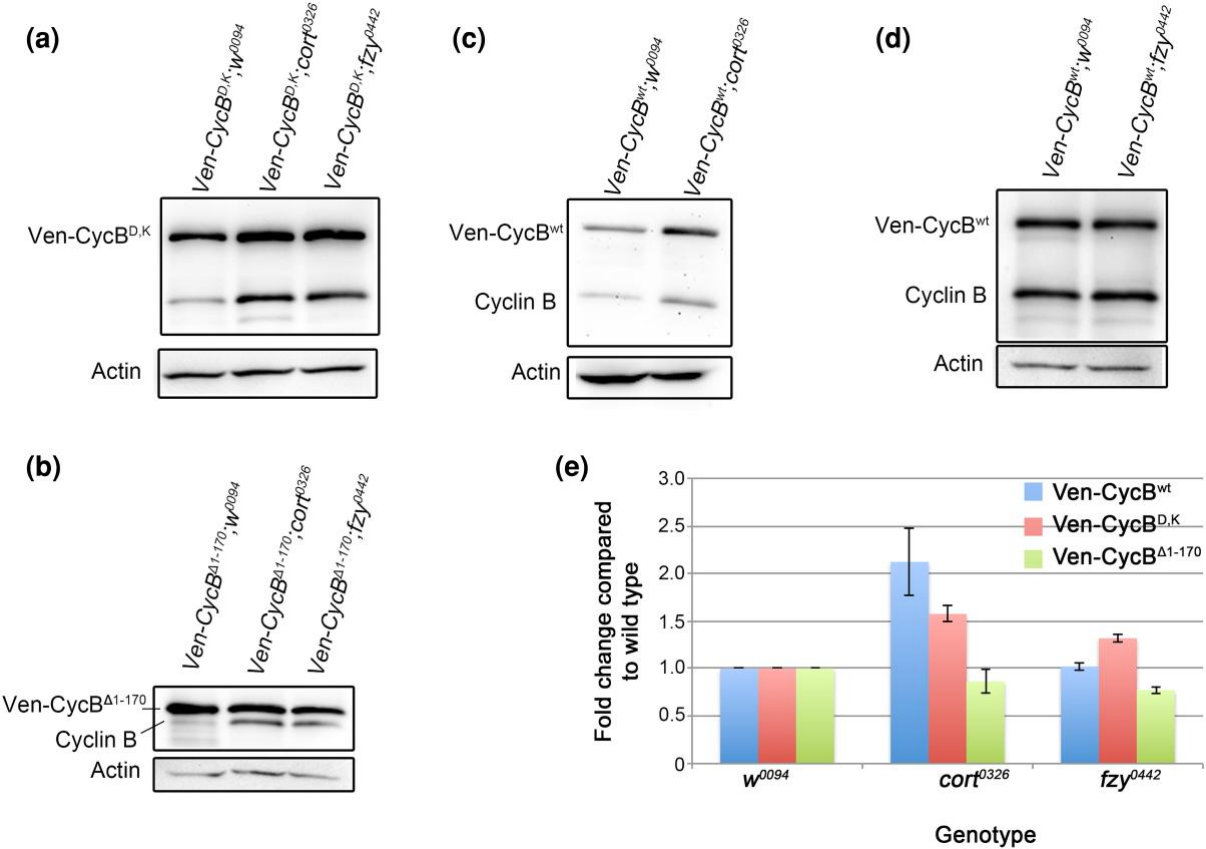
Cort and Fzy target a noncanonical destruction motif on CycB. a–d) *UAS-Venus-CycB* transgenes co-expressed with *UAS-w^0094^* (control) or *UAS-cort*^*0326*^ or *UAS-fzy*^*0442*^ RNAi lines in 0–2 h unfertilized eggs, followed by Western blotting to detect transgenic and endogenous CycB. GFP-CycB^D,K^ levels are elevated in *cort* and in *fzy* knockdown embryos (a). GFP-CycB^Δ1–170^ levels are not elevated in either knockdown (b). GFP-CycB^wt^ levels are elevated in *cort*^*0326*^ (c) but not in *fzy*^*0442*^ (d). e) Quantification of transgenic CycB levels in *UAS-cort*^*0326*^ and *UAS-fzy*^*0442*^ RNAi backgrounds, normalized to transgenic CycB levels in the control, *UAS-w^0094^* background (based on three independent experiments). Error bars represent s.e.m.

**Fig. 7.**
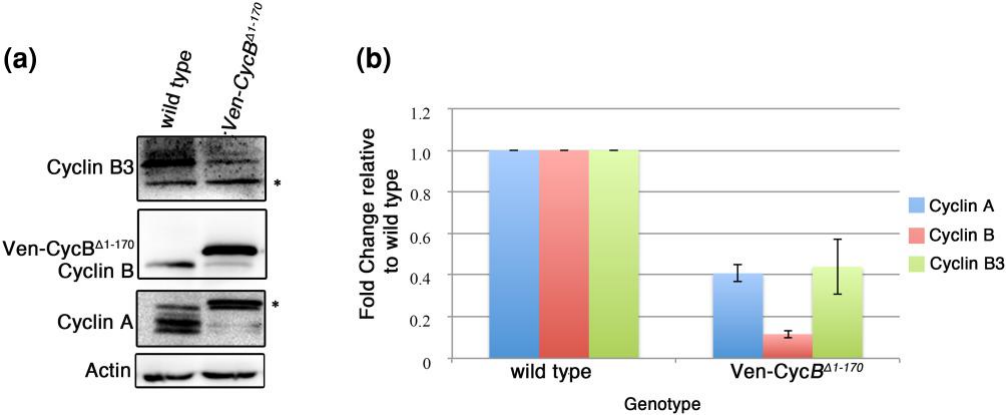
Evidence that Cyclin B can activate APC/C in meiosis. a) Western blot of extracts from wild-type and from *Venus-CycB ^Δ1–170^* eggs probed for the mitotic cyclins. Cyclin A appears as four distinct bands in wild type as described in Vardy *et al.* (2009). Only the lower two bands are apparent in *Venus-CycB*^*Δ1–170*^. Asterisk (*) indicates a nonspecific band. b) Quantification of Cyclin levels from three trials as in (a).

## Results and discussion

### Cyclin A and cyclin B degradation are required for proper completion of meiosis

To determine the consequences of nondegradable cyclins in *Drosophila* female meiosis, we used the maternal Gal4 driver, *matα4-Tubulin-Gal4* to drive female germline expression of UASp Venus (Ven) or GFP-tagged versions of CycA, CycB, and CycB3 that are missing the sequences required for their mitotic destruction. For CycA, we generated two N-terminal deletion mutants based on deletion mutants that were found to be stable in mitotic cells: *UASp-Venus-CycA*^*Δ1–170*^  *(Ven-CycA^Δ1–170^)*, and *Ven-CycA*^*Δ1–53*^ (Parry and O'Farrell 2001; Ramachandran *et al.* 2007). Female germline expression of *Ven-CycA*^*Δ1–170*^ caused oogenesis defects that resulted in failure to obtain late-stage oocytes, and therefore we could not use this line to study meiosis (data not shown). *Ven-CycA*^*Δ1–53*^ also resulted in a reduction in egg production. Examination of developing egg chambers revealed abnormal nurse cell nuclei in which the chromatin was often condensed and associated with arrays of microtubules (Supplementary Fig. 1), suggesting the possibility that these cells had entered an abnormal mitosis. Consistent with this interpretation, chromatin in these nurse cells labeled positive for the mitotic marker, phospho-Histone H1 **(**Supplementary Fig. 1). This phenotype, in which endocycling nurse cells enter into mitosis, is reminiscent of that described for mutants of *APC2* (*morula*), a core APC/C component (Reed and Orr-Weaver 1997). CycA-Cdk1 and APC/C^Fzr^ have mutually inhibitory functions in promoting G2/M and G1, respectively (Dienemann and Sprenger 2004; Reber *et al.* 2006; Hassel *et al.* 2014). It is therefore possible that nondegradable CycA inhibits APC/C^Fzr^ activity in endocycling nurse cells, driving them into a mitosis-like state.

To determine the importance of CycA destruction in meiosis, we first examined the state of the metaphase arrest in mature (stage 14) oocytes. In wild type, mature oocytes are stably arrested in metaphase I, and the bulk of chromatin appears in a single mass (Fig. 1a), with only the small fourth chromosomes oriented toward the poles (not visible in this image). Forty-seven percent of the oocytes from females that express *Ven-CycA*^*Δ1–53*^ (via *matα4-Tubulin-Gal4*) appeared to be arrested in metaphase I with a single major mass of chromatin (Fig. 1a). Thirty percent of the oocytes contained two distinct masses of chromatin, indicative of precocious anaphase (Fig. 1a), while the remaining 22% had a single stretched out mass of chromatin, suggestive of partial segregation of homologues (Fig. 1a). Therefore, stabilized CycA promotes precocious anaphase in stage 14 of *Drosophila* female meiosis.

To determine the effect of nondegradable CycA on further progression of meiosis, we labeled microtubules and chromatin in 0–2 h embryos from wild type and *Ven-CycA*^*Δ1–53*^ females. Wild-type embryos all appeared to be developing and contained multiple synchronized nuclei (Fig. 1b). Embryos from *Ven-CycA*^*Δ1–53*^, by contrast, displayed a complex and variable phenotype. None appeared to develop but instead contained a variable number of spindle-like structures, mainly confined to the dorsal anterior of the egg, where female meiosis normally occurs (Fig. 1c). This complex phenotype suggested that meiosis was disrupted and that embryonic development never occurred. To better understand what happens in meiosis, we collected unfertilized eggs from 0 to 20 and 20–40 min egg lays. In wild type *Drosophila*, the two meiotic divisions occur rapidly, within the first 20 min of egg activation, leading to the formation of four haploid postmeiotic nuclei. If unfertilized, all four nuclei then undergo nuclear envelope breakdown and chromosomes condense and become arranged on an aster of microtubules called a polar body. These often fuse to form one or two larger polar bodies (Page and Orr-Weaver 1997) (Fig. 1d). In a 0–20 min collection from wild-type females, almost all eggs had completed meiosis. Roughly half were in the post-meiotic interphase, while half had formed polar bodies (Fig. 1, d and i). Only a single egg was observed that was in meiosis II, while no meiosis I eggs were observed (Fig. 1, e and i). By contrast, in *Ven-CycA*^*Δ1–53*^ 16% of eggs (*n* = 100) were in meiosis II and 5% were meiosis I (as judged by having 2 or 1 spindle, respectively), indicating that meiosis progression is delayed (Fig. 1i). In all cases, meiosis appeared abnormal—the spindles were mis-shapen and chromatin was not arranged evenly along the spindles as expected for a normal metaphase or anaphase (Fig. 1, f and g). In 43% of eggs, chromatin/microtubule structures were highly aberrant and these eggs could not be categorized with respect to meiotic stage (Fig. 1, h and i). In 20–40 min collections from wild-type females, almost all eggs had completed meiosis and had one or two polar bodies (Fig. 1i). In *Ven-CycA*^*Δ1–53*^ 20–40 min collections, the majority of eggs (88%) had complex phenotypes and could not be classified as belonging to a specific meiotic stage. The complete absence of eggs in post-meiotic interphase, and the low incidence of eggs with polar bodies (Fig. 1i) suggests that meiosis is rarely completed and that the complex microtubule/chromatin arrays that become more common with time represent the effects of aberrant meiosis. It will be important to determine if these defects in meiosis completion are secondary consequences of precocious anaphase, or if they represent distinct requirements for CycA destruction in meiosis.

*Drosophila* CycA levels change dramatically over the course of female meiosis (Vardy *et al.* 2009), and we can now see that this complex regulation is critical for proper meiosis. During the prophase arrest in mid oogenesis, CycA is at very low levels, but it rises dramatically at maturation (Vardy *et al.* 2009). This correlates with the requirement for CycA in nuclear envelope breakdown and the association of homologue pairs with the central spindle, a process that begins shortly after oocyte nuclear envelope breakdown (Bourouh *et al.* 2016). Levels of CycA then drop and remain low during the stage 14 metaphase I arrest (Vardy *et al.* 2009). We propose that APC/C mediates the degradation of CycA during the metaphase I arrest, and that this is necessary to maintain sister chromatid cohesion and thus to maintain this arrest. This may be a conserved process, as the injection of nondegradable CycA in mammalian oocytes also leads to precocious anaphase (Touati *et al.* 2012). It remains to be determined how inappropriate CycA–Cdk1 activity during the metaphase I arrest leads to precocious anaphase, but one possibility is that CycA–Cdk1 is capable of phosphorylating and activating the APC/C at this stage of meiosis.

We next turned to examining the consequences of stabilized CycB on meiosis. Our previous findings with a D-box mutated GFP-CycB^D^ revealed a relatively weak arrest (only 15% of eggs appeared to arrest in meiosis) (Swan and Schupbach 2007). We repeated this experiment using Venus-tagged CycB lacking both D-box and KEN box *(Ven-CycB*^*D,K*^*)*; and with an N-terminus deleted mutant *(Ven-CycB*^*Δ1–170*^*)*. These two stabilized forms of CycB both caused an arrest in meiosis I or more commonly, meiosis II (Fig. 2, a–c). This stronger phenotype could in part be due to higher level expression: Western blots (Figs. 5 and 7, and see *Materials and Methods*) revealed that Ven-CycB^Δ1–170^ is present at 2.6× the level of endogenous CycB (in wild-type eggs), while GFP-CycB^D^ is found at 0.9× endogenous CycB levels. We conclude that stabilized CycB is able to prevent the completion of meiosis, suggesting that APC/C-mediated CycB destruction is necessary for the completion of Drosophila meiosis. This is consistent with results in vertebrates (Herbert *et al.* 2003).

### CycB3 destruction is required for completion of meiosis and for proper microtubule organization

To determine the effect of nondegradable CycB3 on meiosis, we examined 0–2 h embryos from females that expressed a stabilized version of CycB3, GFP-CycB3^D^ (Garrido *et al.* 2020) during oogenesis. Wild-type embryos that are in the early embryonic cell cycles always have one or two polar bodies in the dorsal anterior (Fig. 3a). In contrast, fertilized eggs from *GFP-CycB3*^*D*^ females never had recognizable polar bodies. Most eggs instead contained an array of microtubules with associated chromatin in the location where meiosis normally occurs (Fig. 3b). Distinct microtubule arrays also formed around the cell cortex (Fig. 3 d, d′, compare c, c′). These microtubule arrays were more extensive in older eggs (Supplementary Fig. 2), indicating that microtubule polymerization continued in these eggs even in the absence of embryonic development.

The presence of these microtubule arrays made it impossible to assess whether or not meiosis occurred normally. We, therefore, examined eggs from shorter (0–40 min) collections, and we used FISH against a centromeric region of the X-chromosome to follow the segregation of a single chromosome. Even in these shorter egg collections, microtubule polymerization was apparent, but meiotic spindles could be clearly observed in many cases (Fig. 3f). In others, microtubule polymerization was too extensive to determine meiotic phase by spindle appearance, but the relative spacing of X-chromosomes suggested that they were in meiosis, most commonly, anaphase II (Fig. 3, g and h). In wild-type controls, all eggs had completed meiosis (Fig. 3, e and h).

To determine if this ectopic microtubule polymerization started prior to egg activation, we stained stage 14 oocytes for DNA and microtubules. The meiotic spindle appeared normal and there was no evidence of ectopic microtubules (Fig. 3, i–k). Therefore, nondegradable CycB3 promotes ectopic microtubule formation after egg activation.

Given that CycB3 promotes APC/C activity in meiosis (Garrido *et al.* 2020), we wanted to know if the ectopic microtubules produced by nondegradable CycB3 are due to inappropriate APC/C activation. To test this, we asked if the formation of ectopic microtubules in *GFP-CycB3*^*D*^ eggs depends on the APC/C. We used *cort*, *fzy* double mutants to deplete APC/C activity while expressing *GFP-CycB3*^*D*^. First, we confirmed that *cort*, *fzy* double mutants (not expressing *GFP-CycB3*^*D*^) arrest in meiosis (Swan and Schupbach 2007) with no sign of ectopic microtubule polymerization (Fig. 4, a, a′, c and c′). We then combined the *cort*, *fzy* double mutant with *GFP-CycB3*^*D*^. Eggs from these females invariably arrested in meiosis and showed no indication of ectopic microtubules (Fig. 4, d and d′) (Compare to (Fig. 4, b, b′). Therefore, APC/C activity is necessary for ectopic microtubule formation by GFP-CycB3^D^. We next tested individual *cort* and *fzy* mutants, and found that loss of either gene suppressed the ectopic microtubule phenotype of *GFP-CycB3*^*D*^ (Fig. 4, e–h′). We conclude that nondegradable CycB3 promotes ectopic microtubule polymerization by promoting both APC/C^Cort^ and APC/C^Fzy^ activity.

We next asked if the ectopic microtubule polymerization resulting from stabilized CycB3 is a result of the inappropriate degradation of CycA or CycB by the hyperactive APC/C. To address this question, we asked if the expression of stabilized CycA or CycB could suppress the ectopic microtubule phenotype of *GFP-CycB3*^*D*^. First, we noted that ectopic microtubules were not observed in eggs in which stabilized CycA or stabilized CycB were expressed alone (Figs. 1 and 2, b and c). We then co-expressed these stabilized forms of CycA and CycB with GFP-CycB3^D^. We found that Ven-CycA^Δ1–53^ and Ven-CycB^Δ1–170^ suppressed the microtubule polymerization phenotype of *GFP-CycB3*^*D*^ (Fig. 2, f and g). Interestingly, Ven-CycB^D,K^ did not suppress (Fig. 2e) (we discuss a possible reason below—see Fig. 5). We conclude that stabilized CycB3 leads to ectopic microtubule polymerization by promoting APC/C-mediated degradation of CycB and CycA. If this is true, it implies that CycA–Cdk1 and CycB–Cdk1 complexes regulate microtubule polymerization at the transition from egg to embryo. It will be interesting to see if this relates to the Cort-dependent reorganization of microtubules that normally occurs at egg activation (Page and Orr-Weaver 1996).

### APC/C recognizes a meiosis-specific degron on CycB

We previously observed a dramatic reduction in levels of the mitotic cyclins, CycA, CycB, and endogenous CycB3 upon expression of nondegradable CycB3. Based on other data, we concluded that this effect on cyclin levels was brought about by hyperactivation of the APC/C (Garrido *et al.* 2020). However, it is formally possible that stabilized CycB3 represses cyclin transcription or translation. To distinguish between a role in cyclin degradation and an effect on cyclin expression, we examined the effect of Flag-CycB3^D^ on transgenic CycB, from *UAS-Venus-CycB*^*wt*^ (*Ven*-*CycB*^*wt*^) that does not have endogenous transcriptional or translational regulatory elements. We found that Ven-CycB^wt^ levels were lower when co-expressed with *GFP-CycB3*^*D*^ than when co-expressed with a control *UAS*-*white*^*0994*^ transgene (Fig. 5, a and g). This result supports the conclusion that nondegradable CycB3 promotes APC/C-mediated degradation of CycB, and not transcriptional or translational repression.

Different APC/C activators confer on the complex a different range of substrate specificities. APC/C^Fzy^ specifically recognizes the Destruction box (D-Box—typically RxxLxxxxN). The G1 APC/C^Fzr^ recognizes the D-box and KEN box on cyclins, and some other motifs on other substrates (Yamano 2019). It is not yet known what motifs APC/C^Cort^ can recognize, with the exception of an apparently divergent D-box (LxExxxN) on the meiotic regulator, Matrimony (Whitfield *et al.* 2013). To determine if the APC/C that is activated by *CycB3*^*D*^ recognizes a D-Box on its substrates, *GFP-CycB*^*D*^ (Fig. 5h) (Raff *et al.* 2002) was co-expressed with *Flag-CycB3*^*D*^. Interestingly, GFP-CycB^D^ was efficiently degraded in the presence of Flag-CycB3^D^ (Fig. 5, b and g). Similarly, transgenic CycB that is mutant for the KEN box *(Ven-CycB*^*KEN*^*)* was also efficiently degraded in *Flag-CycB3*^*D*^ eggs (Fig. 5, c and h). We then tested Ven-CycB^D,K^ and found that it was also degraded in the presence of Flag-CycB3^D^ (Fig. 5, d and h).

The degradation of Ven-CycB^D,K^ in *Flag*-*CycB3*^*D*^ eggs indicates that there is another motif that mediates the meiosis-specific degradation of CycB. To identify this sequence we examined the stability of *Ven-CycB*^*Δ1–53,K*^. This deletes the first 53 amino acids (including the D-box) and it is also mutated at the KEN-box. Venus-CycB^Δ1–53,K^ was efficiently degraded in *Flag-CycB3*^*D*^ eggs (Fig. 5, e, g and h). On the other hand, Ven-CycB^Δ1–170^ was stable in the presence of Flag-CyB3^D^ (Fig. 5, f, g and h). This suggests that a destruction motif lies between amino acids 53–170 in the N-terminus of CycB. CycB has a RxxL sequence at residues 162–165 that is not functional as a degron in mitotic cells (Jacobs *et al.* 2001; Kaspar *et al.* 2001; Ramachandran *et al.* 2007). It will be interesting to directly test the significance of this site for the targeting of CycB by the APC/C in meiosis. The finding that Ven-CycB^D,K^ is efficiently degraded in the presence of nondegradable CycB3 could explain why this CycB transgene failed to suppress the *GFP-CycB3*^*D*^ microtubule polymerization phenotype (Fig. 2e).

### Cort and fzy can both recognize the putative meiosis-specific degron on CycB

To identify the specific APC/C activator that targets the putative meiosis-specific degron on CycB, we examined the levels of Ven-CycB^D,K^ in eggs from females depleted of Cort or Fzy. For this experiment, we turned to *UAS-RNAi* transgenes against *cort* and *fzy* due to technical difficulties in combining mutants with the Gal4 driver and stabilized cyclin transgenes. We expressed RNAi against either *cort*, *fzy*, or the *white* gene as a control (*cort*^*0326*^, *fzy*^*0442*^, and *w^0094^*, respectively) together with Ven-CycB^D,K^. Ven-CycB^D,K^ levels were elevated upon *cort* knockdown and to a lesser extent, upon *fzy* knockdown (Fig. 6, a and e). This indicates that both APC/C^Cort^ and APC/C^Fzy^ are able to target CycB that lacks its known D-box and KEN box. In contrast, Ven-CycB^Δ1–170^ levels did not rise in either the *cort* or the *fzy* knockdown background (Fig. 6, b and e), consistent with the idea that Ven-CycB^Δ1–170^ is resistant to APC/C-mediated destruction in meiosis. Also as expected, Ven-CycB^wt^ levels rose in the *cort* knockdown background (Fig. 6, c and e). Unexpectedly, Ven-CycB^wt^ levels did not appear elevated upon *fzy* knockdown (Fig. 6, d and e). This is surprising given that *fzy* mutants lead to elevated CycB levels (Swan and Schupbach 2007) and that *fzy*^*0442*^ produces a slight elevation in levels of Ven-CycB^D,K^. It may reflect an incomplete knockdown from this RNAi transgene. With this caveat, we conclude that APC/C^Cort^, and possibly APC/C^Fzy^ are able to target CycB through a destruction motif that is distinct from the known D-box and KEN box.

### CycB can activate the APC/C

In our experiments examining the stability of Venus-CycB transgenes, we noticed that when stabilized forms of CycB were expressed in an otherwise wild type background, endogenous CycB was only weakly detected (Fig. 5, d and e). This suggested the possibility that nondegradable CycB could, like nondegradable CycB3, stimulate APC/C activation, leading to destruction of endogenous CycB. To test this further, we examined the levels of the three mitotic cyclins in eggs from females expressing *Ven-CycB*^*Δ1–170*^. Expression of *Ven-CycB*^*Δ1–170*^ resulted in a decrease in levels of all three cyclins compared to wild-type eggs, suggesting that CycB can promote APC/C activity (Fig. 7, a and b). Our finding here that CycB can activate the APC/C in meiosis may appear to conflict with the previous finding that CycB inhibits APC/C activity in meiosis I arrested oocytes (Bourouh *et al.* 2016). However, there is precedent for Cdk1–CycB having both positive and negative effects on APC/C^Fzy^ activity. Cdk1-mediated phosphorylation of APC/C core subunits APC3 and APC1 is required for activation of APC/C^Fzy^ (Fujimitsu *et al.* 2016; Qiao *et al.* 2016; Zhang *et al.* 2016). At the same time, Cdk1–CycB-mediated phosphorylation of activator Cdc20 is inhibitory (Labit *et al.* 2012). It is, therefore, possible that in *Drosophila* meiosis Cyclin B has both APC/C inhibiting and activating functions. If this interpretation is correct, it will be interesting to discover how the APC/C-inhibiting and activating functions of CycB–Cdk1 are temporally regulated in meiosis.

Given that nondegradable CycA can promote precocious anaphase, we were also interested in determining if nondegradable CycA leads to increased degradation of the mitotic cyclins. Expression of *Ven-CycA*^*wt*^ or stabilized *Ven-CycA*^*Δ1–53*^ had no effect on levels of CycB, CycB3, or endogenous CycA (Supplementary Fig. 3), indicating that Cdk1–CycA is not able to activate APC/C following egg activation. It will be interesting to repeat these experiments in stage 14 oocytes to see if stabilized CycA promotes APC/C activity at this stage of meiosis.

## Conclusions

By studying the effects of nondegradable cyclins in meiosis we have uncovered distinct functions of the three mitotic Cdk complexes in meiosis. It will be important to follow up these studies by identifying specific substrates that confer on each mitotic Cdk–cyclin complex its unique functions.

## Supplementary Material

jkae066_Supplementary_Data

## Data Availability

*Drosophila* strains and plasmids are available upon request. The authors affirm that all data necessary for confirming the conclusions of the article are present within the article, figures, and tables. Supplemental material available at G3 online.
