# Supplementary material for: Analysis of nondegradable cyclins reveals distinct roles of the mitotic cyclins in Drosophila meiosis
Source: G3 (Bethesda). 2024 Mar 29;14(6):jkae066. doi: 10.1093/g3journal/jkae066 (PMC11152073; doi:10.1093/g3journal/jkae066)
Supplement: jkae066_Supplementary_Data [file jkae066_supplementary_data.docx]

**Supplemental Figures**


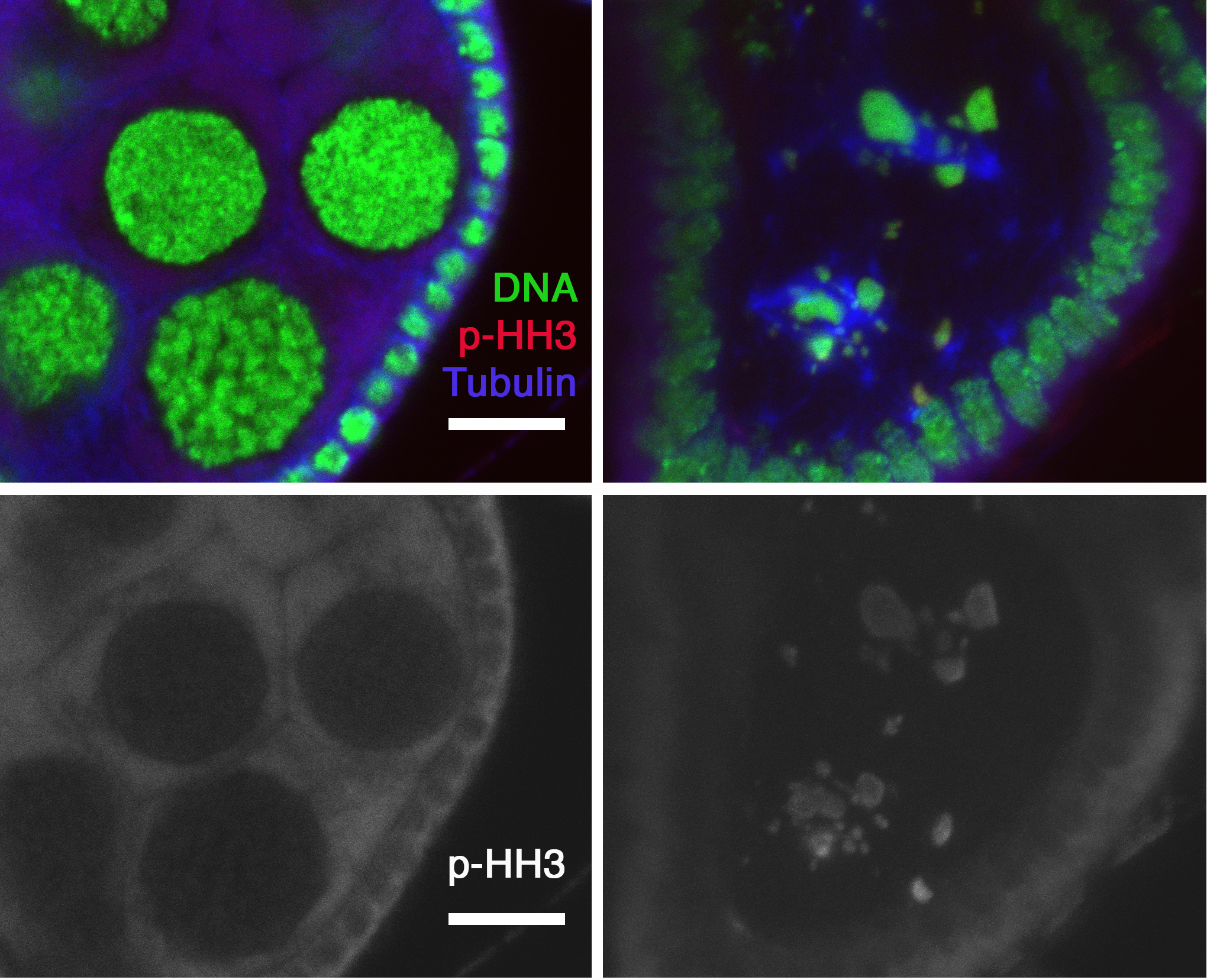


**Figure S1. Non-degradable CycA expression in ovaries disrupts endocycle in nurse cells.**

Egg chambers labeled for chromatin (green), microtubules (blue) and the mitotic marker, phosopho-Histone H3 (red – and grayscale in lower panels). Left, wild type egg chamber. Right, egg chamber from a female expressing *Ven-CycA^Δ1-53^.* Microtubule arrays resembling mitotic figures are associated with condensed, phospho-Histone H3-positive chromatin.


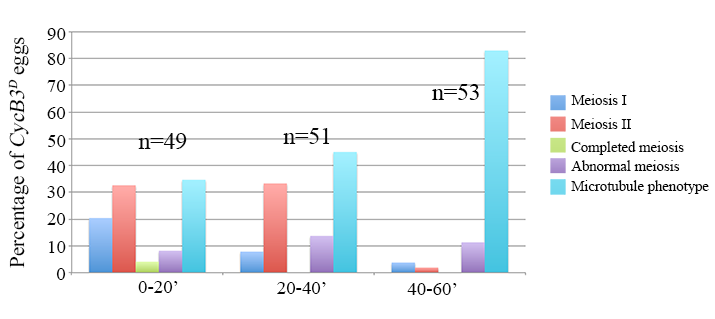


**Figure S2. Microtubule polymerization continues over time in CycB3D eggs.**

Eggs from timed egg lays were stained for microtubules and chromatin and categorized with respect to meioitic phenotype if detectable and microtubule polymerization phenotype. The percentage of eggs with microtubule polymerization increases with age of eggs.


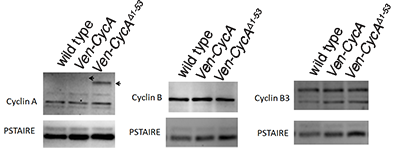


**Figure S3. Stabilized CycA does not promote APC/C activity.**

Western blots to detect levels of CycA, CycB and CycB3 from 0-2 hour unfertilized eggs from wild type or from females overexpressing Venus-CycA or Ven-CycA ^Δ1-53^. Levels of the three cyclins are similar in all cases
